# *Lactobacillus paracasei* N1115 alleviates hyperuricemia in mice: regulation of uric acid metabolism as well as its impact on gut microbiota and short-chain fatty acids

**DOI:** 10.3389/fnut.2025.1651214

**Published:** 2025-10-07

**Authors:** Hongyu Zhang, Da Wang, Dongning Li, Bingkun Bao, Qian Chen, Sunhaoran Wang, Shichao Han, Mengge Zhao

**Affiliations:** ^1^College of Food Science and Engineering, Northwest A&F University, Yangling, Shaanxi, China; ^2^Department of Urology, Xijing Hospital of Air Force Medical University, Xi'an, Shaanxi, China

**Keywords:** *Lactobacillus paracasei*, hyperuricemia, uric acid metabolism, gut microbiota, short-chain fatty acids, *Bifidobacterium*, butyric acid

## Abstract

Hyperuricemia (HUA) is a worldwide metabolic disorder characterized by abnormally elevated serum uric acid (SUA) levels, and recent studies suggest that probiotics have potential in mitigating HUA. This study aimed to evaluate the efficacy of *Lactobacillus paracasei* N1115 in alleviating HUA in mice and explore its underlying mechanisms. The results demonstrated that both high and low-dose *L. paracasei* N1115 reduced SUA levels *in vivo* by 29.18 and 27.29%, respectively (*p* < 0.05), effectively mitigating HUA. Additionally, the probiotic protected renal function, mitigated tissue damages and inflammation. Mechanically, it effected uric acid (UA) metabolism by regulating the UA-production related enzymes xanthine oxidase (XOD), adenosine deaminase (ADA), and 5′-nucleotidase (5′-NT), as well as the urate transpoters urate transporter 1 (URAT1) and glucose transporter 9 (GLUT9), and organic anion transporter 3 (OAT3). Moreover, *L. paracasei* N1115 reshaped the gut microbiota and significantly increased the abundance of *Bifidobacterium*, while modulating renal metabolism and elevating butyric acid levels in gut. These findings suggest that *L. paracasei* may alleviate HUA by enhancing butyrate levels through a cross-feeding interaction with *Bifidobacterium*. Although further experiments are required to substantiate underlying mechanisms, this study provides a basis for HUA-targeting functional foods research.

## Graphical abstract

The image depicts the changes in gut microbiota, metabolites, transport proteins, inflammatory factors, and UA-producing enzymes in HUA mice after intervention with Lactobacillus paracasei N1115. After the intervention, the expression of GLUT9 and URAT1 in the kidneys of the mice decreased, while the expression of OAT3 increased. The abundance of Bifidobacterium in the gut and the level of butyrate rose. Serum indicators showed that after the intervention, the levels of UA, BUN, and Cr decreased, the anti-inflammatory ability was enhanced, and the activity of enzymes related to UA production declined. 
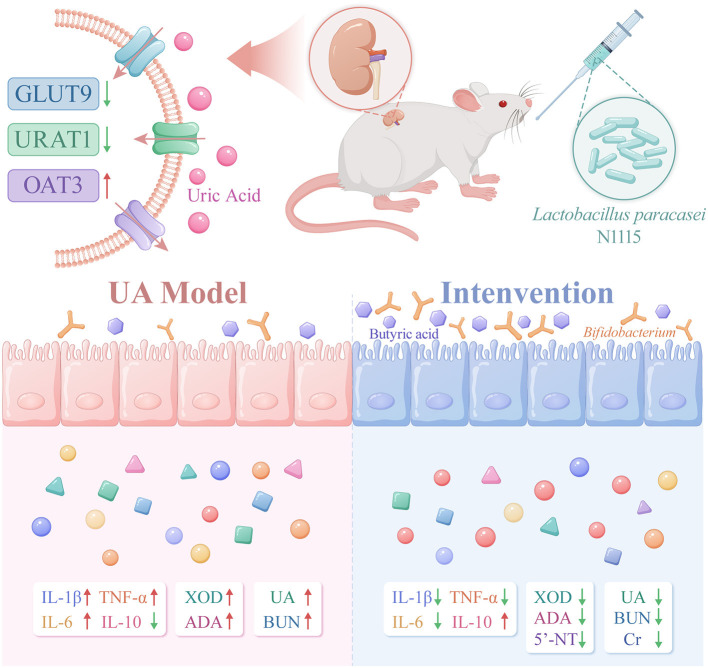

## 1 Introduction

Hyperuricemia (HUA) is a metabolic disorder characterized by abnormally elevated serum uric acid (SUA) levels, primarily resulting from an imbalance between uric acid (UA) production and excretion (1). Recent studies indicate that the overall prevalence of HUA is 19.37%, with 27.72% in men and 10.69% in women, and the incidence of HUA in China has significantly increased, particularly in developed regions (2, 3). HUA is also associated with various diseases, including cardiovascular disease, chronic kidney disease, metabolic syndrome, and type 2 diabetes, etc (4–6). Studies have shown that for every 10 mg/L increase in SUA levels, the risk of hypertension increases by 13%, and HUA as a risk factor can accelerate the progression of atherosclerosis and heart failure (7). In addition, when SUA are too high, crystalline precipitation occurs, which can lead to complications such as gout in joints and cartilage areas (8). Therefore, patients with HUA need to take effective measures to reduce SUA.

With ongoing research into the anti-HUA functional foods, probiotics have emerged as a promising strategy for HUA intervention. Recent studies have increasingly demonstrated a significant association between gut microbiota and the progression of HUA, and probiotics have shown potential therapeutic value through the multi-target regulation of UA metabolism. Specifically, certain probiotics, such as *Levilactobacillus brevis* grx821, competitively inhibit the absorption of exogenous purines in the gut, thereby reducing the accumulation of precursors for UA synthesis and lowering SUA levels (9). Additionally, probiotics can modulate the gut microbiota composition, restore microbial balance, enhance intestinal barrier function, which can promote UA metabolism (10, 11). In terms of anti-inflammatory and immune modulation, some probiotics can alleviate systemic inflammatory responses induced by HUA, which in turn helps improve HUA-related metabolic syndrome (12). Metabolites of probiotics, including short-chain fatty acids (SCFAs), polyamines, and indole derivatives, directly or indrectly participate in UA metabolism. For instance, acetate, a type of SCFAs, can inhibit XOD activity, while butyrate has been widely recognized for its protective effect on the intestinal mucosal barrier and HUA remittence (13, 14). Probiotics can also influence the expression of urate transporters in the gut and kidneys, thereby enhancing UA excretion (15). Moreover, probiotics can treat HUA by affecting other metabolic pathways in the host. For example, *Lactobacillus rhamnosus* UA260 and *Lactobacillus plantarum* YU28 can mitigate HUA via regulating tryptophan metabolism, particularly the production of indole-3-lactic acid (IPA) and indole-3-acetic acid (IAA), which were correlated with XOD activity, colonic injury, and the expression of the UA transporter protein ATP-binding Cassette Sub-family G Member 2 (ABCG2) during treatment (16). Therefore, probiotics are considered to exert their effects on HUA through multiple mechanistic targets. Such multifaceted actions highlight their potential as natural candidates for the functional foods with anti-HUA properties.

Recently, numerous studies have demonstrated the UA-lowering ability of certain lactic acid bacteria. In this study, we assessed the anti-HUA effect of *L. paracasei* N1115 through the *in vitro* and *in vivo* experiments of HUA mouse model. Additionally, we aimed to elucidate the possible mechanisms underlying its ability to mitigate HUA through analyses of HUA associated biomarkers, histopathological examination, urate-producing enzymes, transporter protein expression, gut microbiota composition, renal metabolism and SCFAs.

## 2 Materials and methods

### 2.1 *In vitro* experiments

#### 2.1.1 Strain cultivation and supernatant collection

Methods were as previously described, with slight modifications (16). *L. paracasei* N1115 (from Junlebao Dairy Co., Ltd., Hebei, China) was selected and activated. The cultures were incubated statically at 37 °C for 24 h. Subsequently, 2 ml of each culture was centrifuged at 12,000 g and 4 °C for 10 min, and the supernatant was collected for the XOD inhibition assay.

#### 2.1.2 XOD activity inhibition assay

A centrifuge tube was sequentially loaded with 550 μl of PBS buffer (pH 7.4), 50 μl of the sample, and 200 μl of 0.2 U/ml XOD solution. Before mixing, the pH of the supernatant was adjusted to neutral. The mixture was thoroughly mixed, followed by the addition of 200 μl of 0.2 g/L xanthine solution. The XOD and xanthine solutions were preheated to 37 °C prior to use. The mixture was incubated for 5 min, with absorbance measured at 295 nm at 1-min intervals. Linear regression was performed to determine the slope k_1_. For the blank control, 50 μl of MRS was added in place of the sample, and the corresponding slope k_0_ was determined. The relative inhibition rate of XOD was calculated using formula (1). Methods were as previously described, with some modifications (16).


(1) $$\begin{array}{l} \text{XOD activity inhibition rate }=\frac{{\text{k}}_{0}\;-\;{\text{k}}_{1}}{{\text{k}}_{0}}\times 100\text{\% } \end{array}$$

### 2.2 Animal experiments

#### 2.2.1 Animal experimental design

**Figure 1:**
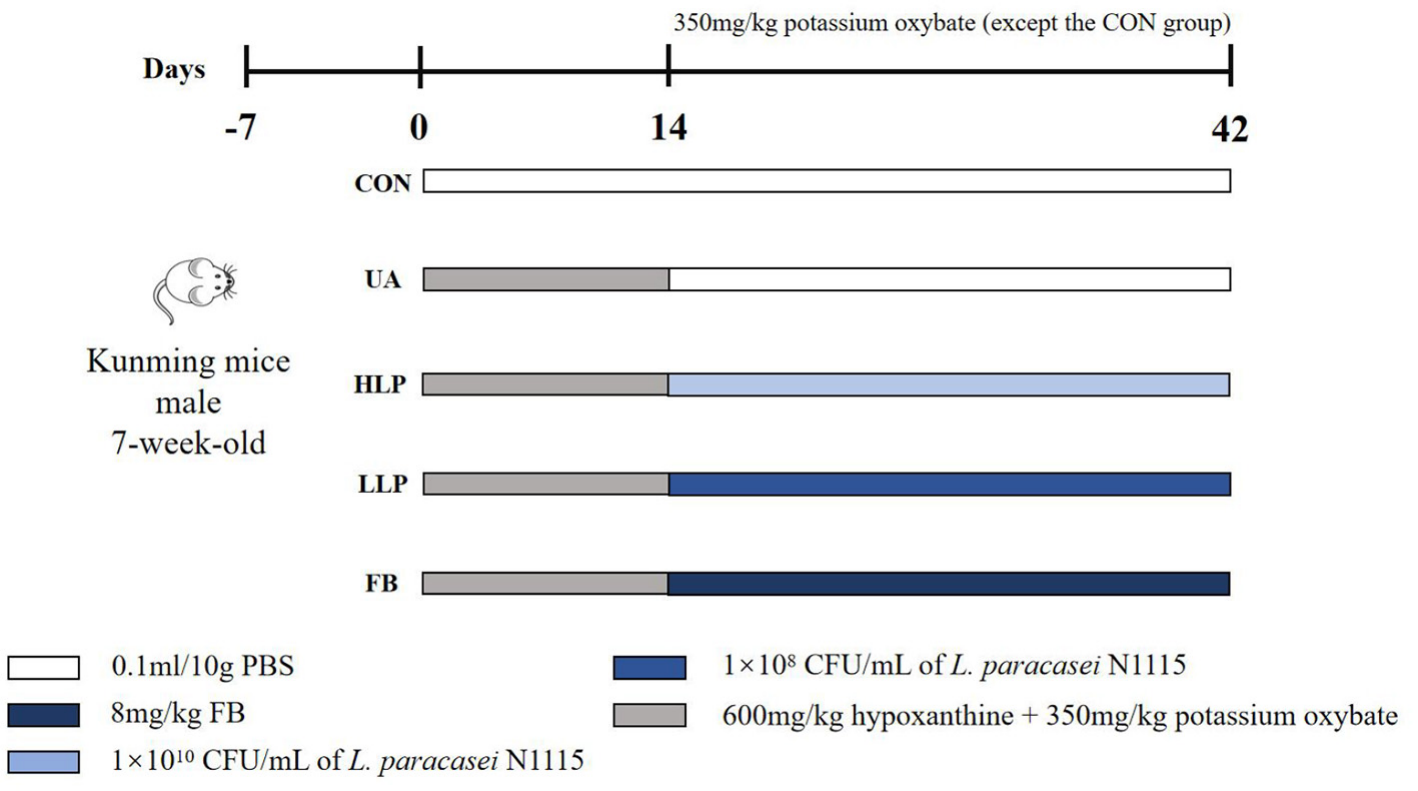
Animal experimental groups and respective treatments. From days 0 to 14, all groups, except the CON group, were orally administered 600 mg/kg of hypoxanthine and 350 mg/kg of potassium oxonate by gavage each day. From days 14 to 42, the UA, HLP, LLP, and FB groups discontinued hypoxanthine administration. The HLP and LLP groups were administered *L. paracasei* N1115 by gavage at concentrations of 1 × 10^10^ CFU/ml and 1 × 10^8^ CFU/ml per day, respectively. The FB group was administered 8 mg/kg of febuxostat daily. Potassium oxonate (350 mg/kg) continued to be administered to the UA, HLP, LLP, and FB groups once every 3 days. Potassium oxonate and the anti-hyperuricemia interventions were administered at intervals of no less than 8 h.

Specific pathogen-free (SPF) Kunming mice (7-week-old, male, *n* = 30) were obtained from Si Pei Fu (Beijing) Biotechnology Co., Ltd. [Animal Qualification Certificate No. SCXK (Beijing) 2019-0004, Beijing, China]. At the end of the acclimatization period, the mice were randomly divided into five groups: control group (CON group), HUA model group (UA group), high-dose *L. paracasei* N1115 group (HLP group), low-dose *L. paracasei* N1115 group (LLP group), and febuxostat group (FB group). The doses and treatments for each group are shown in Figure 1. During the first 2 weeks, all groups, except the CON group, were administered 600 mg/kg of hypoxanthine and 350 mg/kg of potassium oxonate via gavage daily. In the 3rd week, the UA, HLP, LLP, and FB groups ceased hypoxanthine intervention. The HLP and LLP groups were gavaged 1 × 10^10^ CFU/ml and 1 × 10^8^ CFU/ml of *L. paracasei* N1115 per day, respectively (17). The FB group daily received 8 mg/kg of febuxostat. Potassium oxonate (350 mg/kg) continued to provide in UA, HLP, LLP, and FB groups once every 3 days to inhibit uricase in mice, as uricase can convert UA into allantoin (18, 19). The potassium oxonate and anti-HUA interventions were administered at intervals of at least 8 h. All animals used in this study were maintained under SPF conditions with a temperature of 18–24 °C and humidity of 35%−60% and 12 h light/dark cycle and free access to food and water. Reagents used in the animal experiments were purchased from Shanghai Aladdin Biochemical Technology Co., Ltd. (Shanghai, China).

At the end of the experiment (day 42), the mice were fasted for 12 h, anesthetized with isoflurane, and then euthanized. Feces, organs, and blood were collected for subsequent analyses. Serum was obtained by centrifuging the collected blood samples. The research methods were approved by the Animal Ethics Committee of Northwest A&F University (Certificate No.: SCXK [SHAAN] 2017-003; Approval Number: IACUC2024-0715).

#### 2.2.2 Weights and organ coefficients

Weights were measured and recorded weekly. The weights of the liver and kidneys were also recorded, and organ coefficients were calculated using the following formulas:


(2) $$\begin{array}{l} \text{Liver coefficient}=\frac{\text{Liver weight}}{\text{Body weight}}\;\times 100\text{\% } \end{array}$$

(3) $$\begin{array}{l} \text{Kidney coefficient }=\;\frac{\text{Kidneys weight}}{\text{Body weight}}\times 100\text{\% } \end{array}$$

### 2.3 Post-mortem analyses

#### 2.3.1 Serum analysis

The kits were used to measure the levels of SUA, blood urea nitrogen (BUN), creatinine (Cr), xanthine oxidase (XOD), adenosine deaminase (ADA; Beijing Solarbio Science & Technology Co., Ltd., Beijing, China), and 5′-nucleotidase (5′-NT; Jiangsu Meibiao Biotechnology Co., Ltd., Jiangsu, China) in serum, following the manufacturer's instructions.

#### 2.3.2 Inflammatory factors

Protein levels of interleukin-1β (IL-1β), interleukin-6 (IL-6), tumor necrosis factor-α (TNF-α), and interleukin-10 (IL-10) were measured using ELISA kits (Jiangsu Meibiao Biotechnology Co., Ltd., Jiangsu, China), following the manufacturer's instructions.

#### 2.3.3 Histopathological examination

After euthanizing the mice, the kidneys, liver, and duodenum were harvested and processed into H&E-stained sections by Wuhan Servicebio Technology Co., Ltd. (Hubei, China). The stained sections were examined under a microscope (M205FCA, Leica, Germany) and photographed.

#### 2.3.4 Immunohistochemistry and immunofluorescence

The sections were prepared as previously described, with minor modifications (16, 20). The tissue sections were deparaffinized, rehydrated, permeabilized, and subjected to antigen retrieval. After blocking with 3% hydrogen peroxide (H_2_O_2_), the sections were incubated with primary antibodies overnight. Immunohistochemical staining was performed on sagittal kidney sections using antibodies specific for urate transporter 1 (URAT1), organic anion transporter 1 (OAT1), organic anion transporter 3 (OAT3), and nucleoside phosphate transporter 1 (NPT1). After primary antibody incubation, the sections were incubated with a secondary antibody conjugated to a histochemical reagent, followed by DAB staining after hematoxylin counterstaining. Immunofluorescence staining for glucose transporter 9 (GLUT9) was conducted on sagittal kidney sections. These sections were incubated with primary antibodies, followed by incubation with a fluorescent secondary antibody and DAPI staining. The sections were then sealed with an antifluorescence quenching agent. Observations were made using a fluorescence microscope (M205FCA, Leica, Germany), and quantitative analysis was performed using ImageJ software. All antibodies were purchased from Affinity Biosciences Co., Ltd. (Jiangsu, China).

#### 2.3.5 16S rRNA gut microbiota profiling

After the mice were anesthetized and euthanized, fresh feces were collected from the intestines using sterile forceps and stored at −80 °C for subsequent microbiological analysis (*n* = 4). Genomic DNA was extracted from the samples using the CTAB/SDS method, and the variable region (V3-V4) of the 16S rRNA gene was amplified with the forward primer 338F (ACTCCTACGGGGAGGCAGC) and the reverse primer 806R9 (GGACTACHVGGGGTWTCTAAT). The libraries were sequenced on the appropriate platforms to obtain the sequences. The experiment was conducted by Biomarker Technologies Co., Ltd. (Qingdao, Shandong), and the data were analyzed using BMKCloud (http://www.biocloud.net).

#### 2.3.6 Renal non-targeted metabolomics

Samples were analyzed using an Ultra-Performance Liquid Chromatography system coupled with a time-of-flight mass spectrometer. Four samples were randomly selected from each group for omics analysis. A 2.1 mm × 100 mm, 1.7 μm BEH Amide column, maintained at 40 °C, was used to separate the analytes before mass spectrometric analysis. The mass spectrometer was operated in positive ion electrospray ionization full scan mode. Automatic calibration of the spectral peaks was performed to correct any deviations in the lock mass. The experiment was conducted by BestMs Technologies Co., Ltd. (Qingdao, Shandong), and analysis was performed using BestMsCloud (http://www.bestms.cn).

#### 2.3.7 SCFAs concentration analysis

The concentration of SCFAs was determined as previously described (21). Fecal water was prepared by homogenizing the fecal samples with MilliQ water (1 ml) for 10 min using a vortex mixer. Diethyl ether (1.6 ml) and H_2_SO_4_ (50%, w/w) were added to the samples, followed by 20 min of oscillation on a table concentrator at 4 °C. The mixture was then centrifuged at 15,000 g for 5 min (4 °C), and the supernatant was filtered through 0.2 μm filters to remove bacteria and other solids. It was transferred to a 250 μl insert, which was placed in a 2 ml GC vial. The SCFAs of samples were analyzed by using gas chromatograph (Japan, Shimadzu Corporation, GC-2014C), and 2-ethylbutyric acid (Sigma) was used as the internal standard. A standard curve was constructed as well, and Varian Star Chromatography Workstation (version 6.0) was performed to analyze the peak profiles.

### 2.4 Statistical analysis

All data are presented as mean ± SD. Statistical differences between each group were assessed for significance using one-way analysis of variance (ANOVA). All statistical analyses were conducted using GraphPad Prism (version 8.0). *p*-values less than 0.05 were considered statistically significant.

## 3 Results

### 3.1 *L. paracasei* N1115 reduced SUA and improved serum biochemical indicators

**Figure 2:**
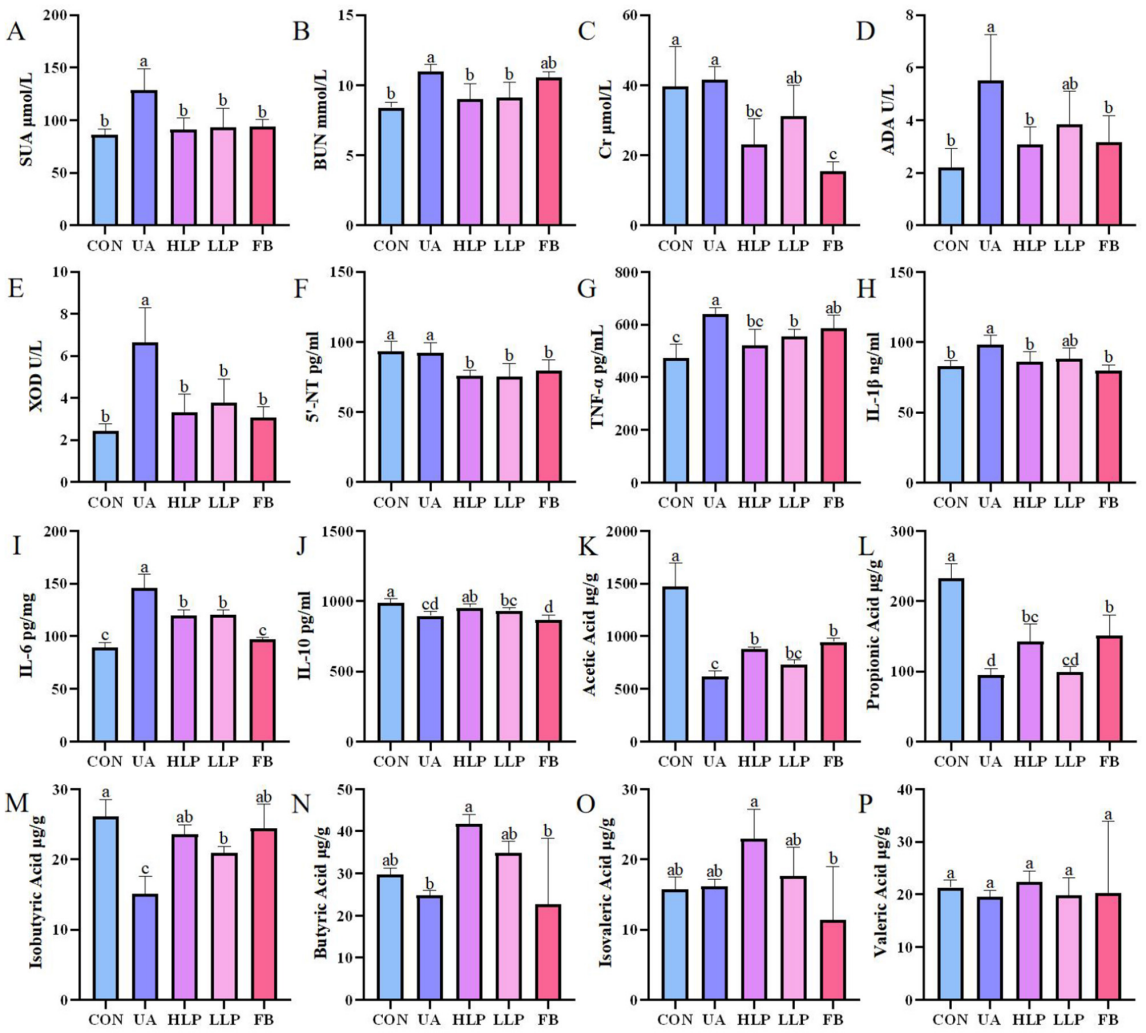
*L. paracasei* N1115 regulated UA metabolism and SCFAs. **(A)** SUA levels, **(B)** BUN levels, **(C)** Cr levels, **(D)** ADA activities, **(E)** XOD activities, **(F)** 5′-NT levels, **(G)** TNF-α levels, **(H)** IL-1β levels, **(I)** IL-6 levels, and **(J)** IL-10 levels in serum (*n* = 6), as well as **(K)** acetic acid levels, **(L)** propionic acid levels, **(M)** isobutyric acid levels, **(N)** butyric acid levels, **(O)** isovaleric acid levels, and **(P)** valeric acid levels in feces (*n* = 4). Values are expressed as the means ± SD. Different letters marked above the bar indicate significant difference (*p* < 0.05).

The SUA level in the UA group was significantly higher than the CON group by 49.43% (*p* < 0.05; Figure 2A), confirming successful establishment of the HUA mouse model. After *L. paracasei* N1115 intervention, SUA levels were significantly reduced in both high and low-dose groups compared with the UA group, deceasing by 29.18 and 27.29%, respectively (*p* < 0.05), comparable to the CON and FB groups (*p* > 0.05). BUN and Cr levels are considered to be indicators of renal function (22). The BUN level in the UA group was significantly higher than that in the CON group (*p* < 0.05; Figure 2B), indicating impaired renal function. Following probiotic intervention, BUN levels in both HLP and LLP groups returned to the normal level. Although, the BUN in the FB group was reduced, the difference compared with the UA group was not statistically significant (*p* > 0.05). Additionally, both high-dose *L. paracasei* N1115 and Febuxostat effectively reduced serum Cr levels (Figure 2C). These findings suggest that *L. paracasei* N1115 is effective in regulating SUA levels in mice and may offer potential renal protection.

### 3.2 *L. paracasei* N1115 inhibited UA metabolism enzymes

Enzymes such as 5′-NT, ADA, and XOD play crucial roles in the pathway of UA synthesis (23). *In vitro* experiments showed that the metabolic products of *L. paracasei* N1115 inhibited XOD activity by 62.14%, demonstrating a strong inhibitory effect. The activities of ADA, XOD, and the content of 5′-NT in serum were also measured to investigate the effect of *L. paracasei* N1115 on UA metabolizing enzymes in mice. The results showed that the activities of ADA and XOD were significantly elevated in the UA group compared with the CON group (*p* < 0.05; Figures 2D, E). Both ADA and XOD activities were significantly inhibited in the HLP group, with reductions of 44.00 and 49.95%, respectively (*p* < 0.05), and were comparable to the FB group (*p* > 0.05). The inhibitory ability of ADA and XOD in the LLP group was weaker than those in the HLP group, but the difference was not statistically significant (*p* > 0.05). Regarding 5′-NT, no significant difference was observed between the CON and UA groups (*p* > 0.05; Figure 2F), suggesting that hypoxanthine and potassium oxonate had little effect on 5′-NT level. However, compared with CON and UA groups, both *L. paracasei* N1115 and febuxostat were able to reduce 5′-NT levels (*p* < 0.05), which may be vital for lowering SUA levels.

### 3.3 *L. paracasei* N1115 ameliorated systemic inflammation

Subsequently, the protein levels of IL-10, IL-6, IL-1β, and TNF-α were measured to analyze the effects of *L. paracasei* N1115 on inflammatory factors. The results showed that the pro-inflammatory factors TNF-α (Figure 2G), IL-1β (Figure 2H), and IL-6 (Figure 2I) were significantly increased in serum and the anti-inflammatory factor IL-10 (Figure 2J) was significantly decreased in the UA group compared with the CON group (*p* < 0.05). Both the HLP and LLP groups exhibited a significant reduction in the levels of TNF-α, IL-1β, and IL-6, along with a modest increase in IL-10. Specifically, compared with UA group, the TNF-α levels in the HLP and LLP groups decreased by 18.40% (*p* < 0.05) and 13.22% (*p* < 0.05); IL-1β decreased by 12.52% (*p* < 0.05) and 10.06% (*p* > 0.05); IL-6 decreased by 22.68% (*p* < 0.05) and 21.67% (*p* < 0.05); and IL-10 increased by 6.55% (*p* < 0.05) and 4.18% (*p* > 0.05), respectively. The observed increase in anti-inflammatory capacity is of significant importance for HUA mice. Febuxostat was also effective in reducing the pro-inflammatory cytokines IL-1β and IL-6 (*p* < 0.05), but the level of the anti-inflammatory cytokine IL-10 decreased rather than increased, indicating that its anti-inflammatory effect was less pronounced than that of *L. paracasei* N1115.

### 3.4 *L. paracasei* N1115 alleviated weight loss and structural damage in the kidney, liver, and duodenum induced by HUA

**Figure 3:**
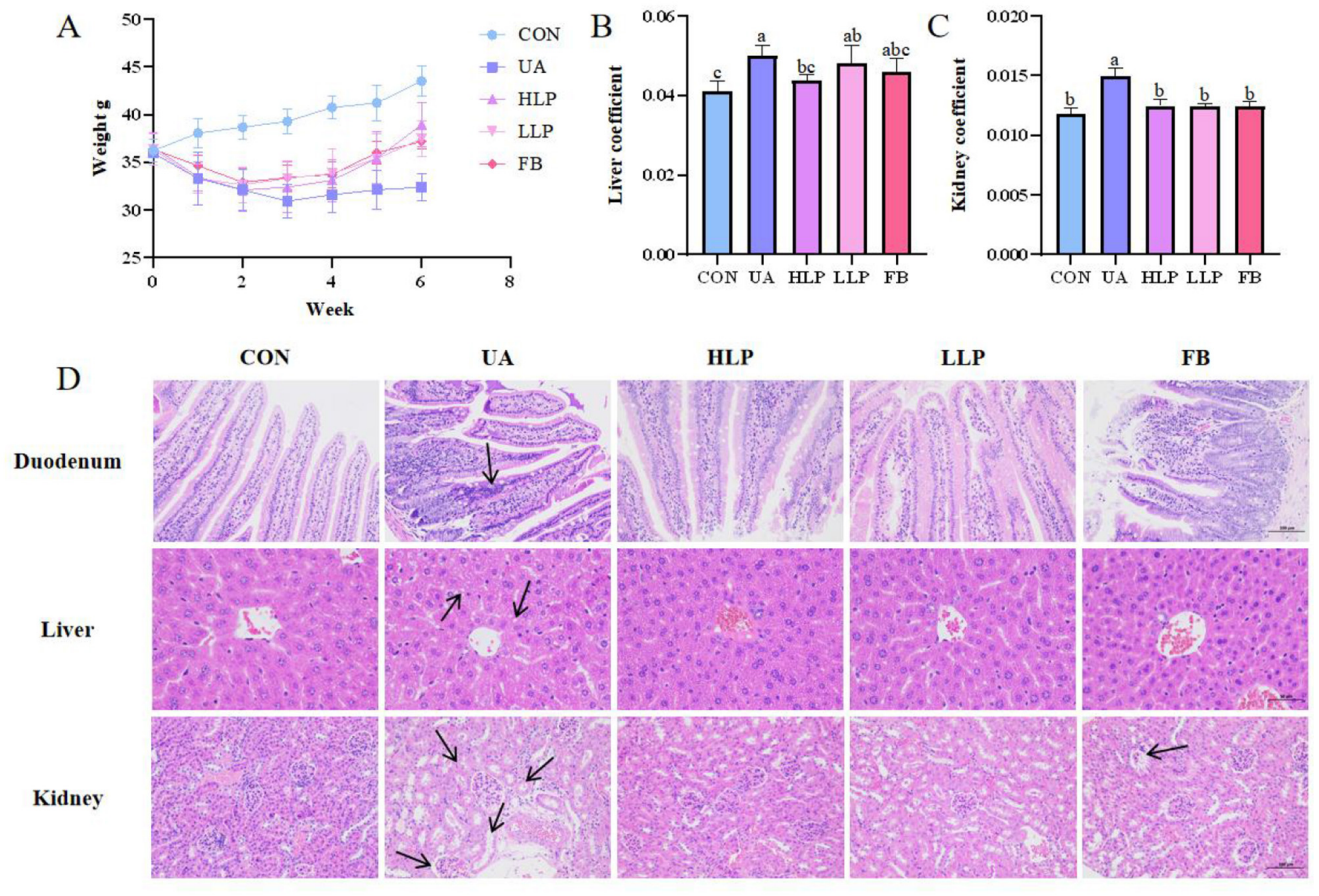
Effects of *L. paracasei* N1115 on weight and tissue damages in HUA mice. **(A)** Weight line. **(B)** Liver coefficient. **(C)** Kidney coefficient. **(D)** Representative images of H&E staining in the duodenum ( × 200), liver ( × 400), and kidney ( × 200). Values are expressed as the means ± SD (*n* = 6). Different letters marked above the bar indicate significant difference (*p* < 0.05).

By measuring body weight and coefficients on liver and kidney in mice, it was found that *L. paracasei* N1115 was effective in mitigating weight loss induced by HUA (Figure 3A). Liver coefficients and kidney coefficients were significantly increased in the UA group compared with the CON group (*p* < 0.05; Figures 3B, C), and the intervention of febuxostat and probiotics significantly reversed these indices. To assess the effect of probiotics on tissue structural damage under HUA conditions, pathological sections of liver, kidney and duodenum were examined (Figure 3D). In the UA group, small intestinal villi were shortened, accompanied by noticeable inflammatory infiltration. Additionally, a significant reduction in goblet cells was observed in FB group. After intervention with *L. paracasei* N1115, inflammation was alleviated, and villus length was restored. In the CON group, the morphology of hepatocytes was normal, while the UA group exhibited numerous vacuoles. In contrast, the HLP group, LLP group, and FB group demonstrated significant improvement. Renal tubules in the UA group were markedly dilated, along with glomerular atrophy and cellular edema, while the FB group displayed severe glomerular deformation. *L. paracasei* N1115 ameliorated the aforementioned adverse symptoms.

### 3.5 *L. paracasei* N1115 affected the expression of renal urate transporters

**Figure 4:**
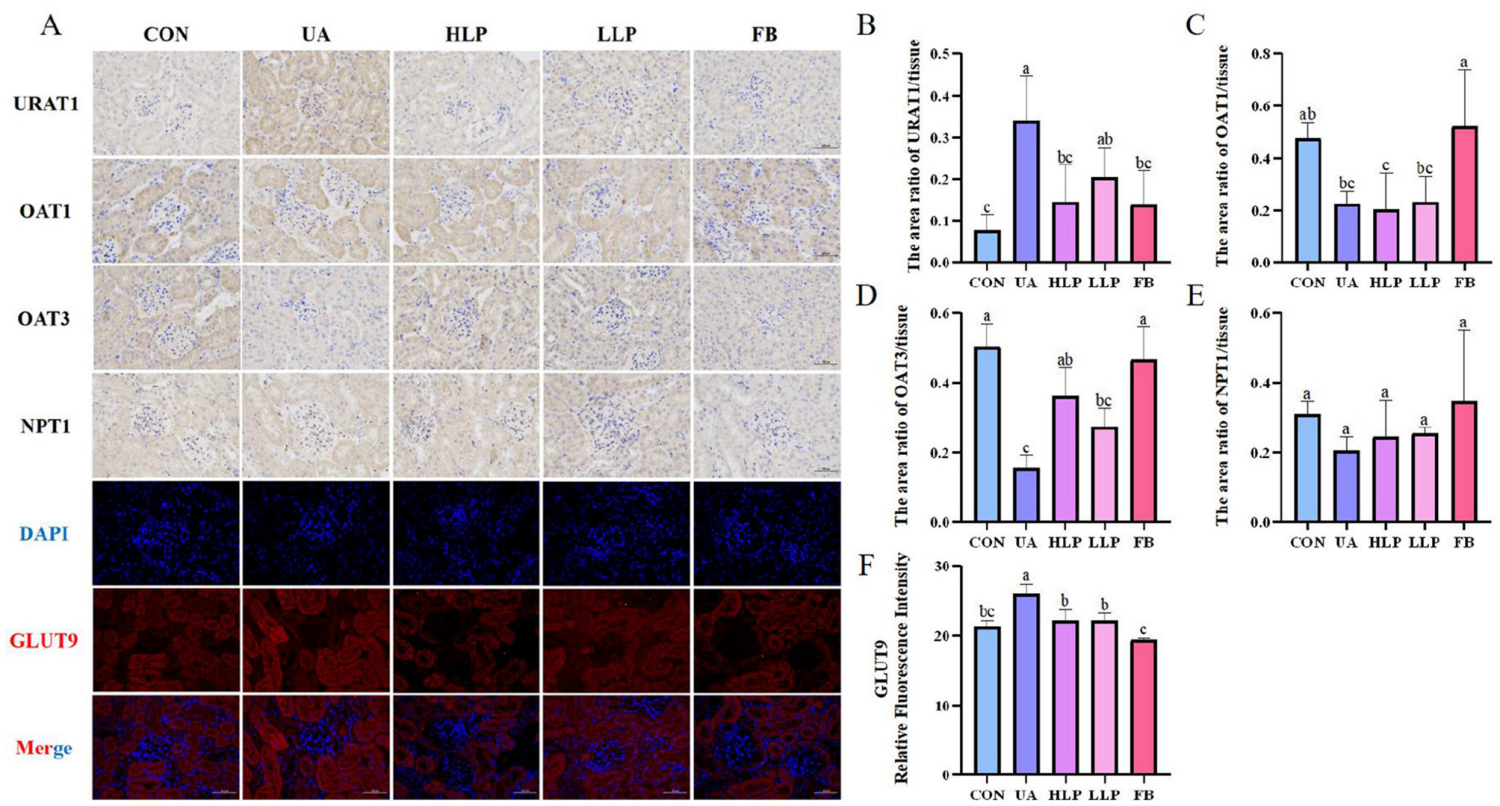
Effects of *L. paracasei* N1115 on renal excretory and reabsorptive transporter proteins. **(A)** Representative images ( × 400) of URAT1, OAT1, OAT3, NPT1 proteins immunohistochemistry, and GLUT9 protein immunofluorescence. Blue and red letters represent DAPI and GLUT9, respectively. **(B)** The area ratio of URAT1/tissue. **(C)** The area ratio of OAT1/tissue. **(D)** The area ratio of OAT3/tissue. **(E)** The area ratio of NPT1/tissue. **(F)** The relative fluorescence intensity of GLUT9. Values are expressed as the means ± SD (*n* = 4). Different letters marked above the bar indicate significant difference (*p* < 0.05).

Next, the levels of URAT1, GLUT9, OAT1, OAT3, and NPT1 were measured to investigate the effects of *L. paracasei* N1115 on renal transporters in mice. As shown in Figure 4, compared with the CON group, the expression of URAT1 and GLUT9 was significantly elevated in the UA group (*p* < 0.05; Figures 4B, F), while the expression of OAT1 and OAT3 was significantly reduced (*p* < 0.05; Figures 4C, D). However, there was no significant change in the expression of NPT1 (*p* > 0.05; Figure 4E). After the intervention of *L. paracasei* N1115, the expression of URAT1 decreased by 57.27% (*p* < 0.05) and 39.94% (*p* > 0.05) in the HLP and LLP groups, respectively, with no significant difference from the FB group (*p* > 0.05). Similarly, GLUT9 expression decreased by more than 14% in both high and low-dose groups (*p* < 0.05). In addition, excretory transporter protein OAT3 increased by 136.04% (*p* < 0.05) and 78.08% (*p* > 0.05) in the HLP and LLP groups, respectively. Febuxostat showed superior regulatory effects on OAT3 and GLUT9 compared with *L. paracasei* N1115. Unfortunately, no significant effects of *L. paracasei* N1115 on OAT1 and NPT1 was observed (*p* > 0.05). These findings suggest that *L. paracasei* N1115 may promote UA excretion by modulating the reabsorption transporter proteins URAT1 and GLUT9, as well as the excretion transporter protein OAT3.

### 3.6 *L. paracasei* N1115 remodeled intestinal microbiota structure in HUA mice

**Figure 5:**
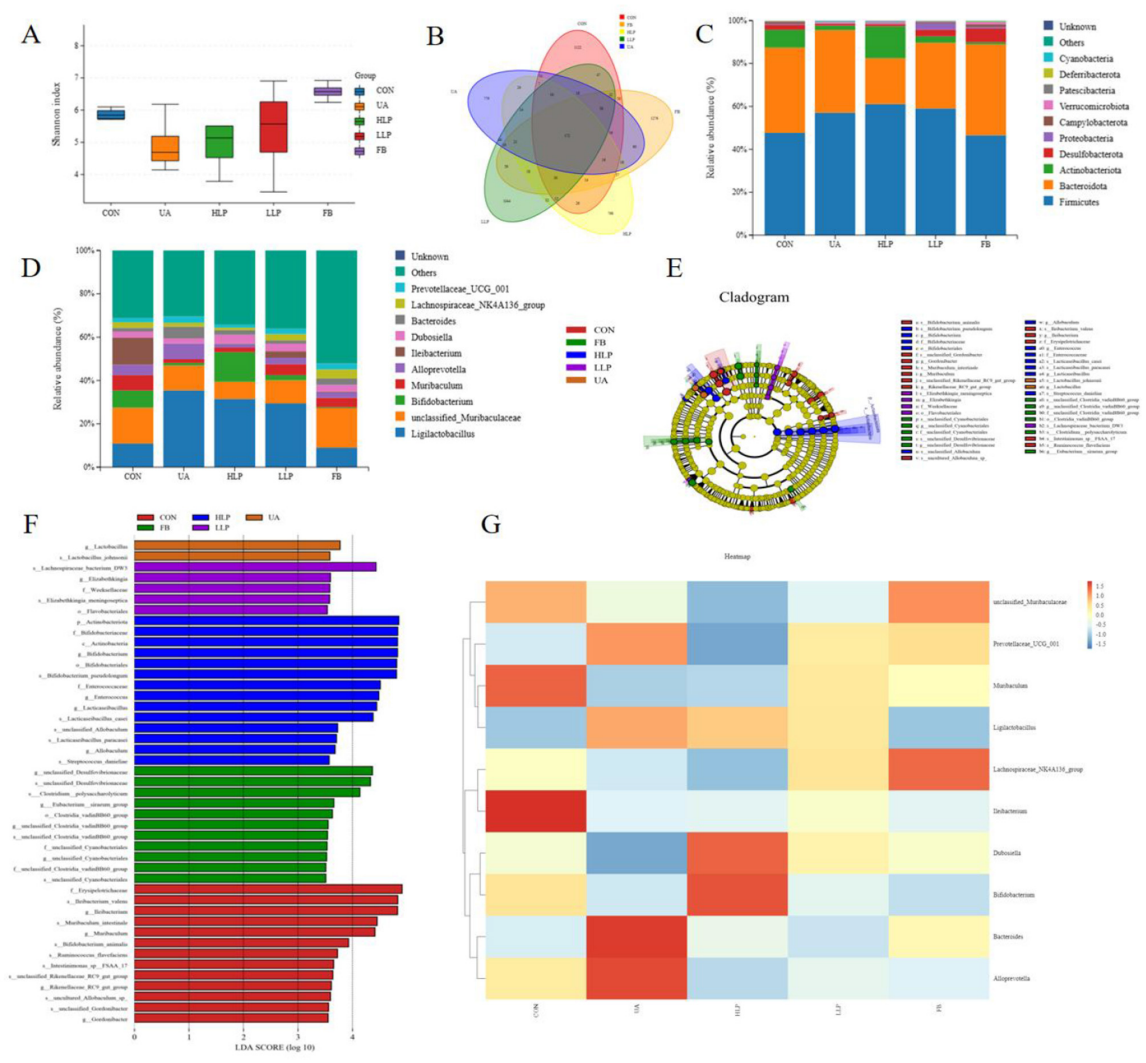
Effect of *L. paracasei* N1115 on the structure of the gut microbiota in HUA mice. **(A)** Shannon index. **(B)** Venn diagram. **(C)** Bar graphs showing the relative abundance of different bacteria at the phylum level. **(D)** Bar graphs showing the relative abundance of different bacteria at the genus level (*n* = 6). **(E)** The cladogram generated from LEfSe analysis. **(F)** Diagram of significantly different species with LDA score greater than 3.5. **(G)** Heat map depicting the composition of gut microbiota species. Data are expressed as mean ± SD (*n* = 4).

In this study, 16S rDNA region 3 and 4 sequencing analysis was performed to understand the effects of *L. paracasei* N1115 on the gut microbiota. Shannon's index indicated that exposure to hypoxanthine and potassium oxonate reduced the overall microbial community diversity. After treatment with *L. paracasei* N1115, species richness increased, with a slightly higher value in the LLP group compared with the HLP group (Figure 5A). Statistical analysis of operational taxonomic units (OTUs), displayed in the Venn diagram, revealed significant differences in composition between the groups (Figure 5B).

The species relative abundance of the gut microbiota was analyzed as well. At the phylum level, the CON group predominantly featured *Firmicutes* (47.70%), *Bacteroidota* (39.69%), *Actinobacteriota* (8.33%), *Desulfobacterota* (2.34%), and *Campylobacterota* (1.01%). The relative abundance of microbial communities in the UA group changed significantly, with a decrease in *Actinobacteriota* (2.10%), *Desulfobacterota* (0.93%), and a significant increase in *Firmicutes* (57.10%). Following intervention with *L. paracasei* N1115, *Firmicutes* increased moderately (HLP group: 61.11%; LLP group: 59.04%) and *Bacteroidota* decreased substantially (HLP group: 21.33%; LLP group: 30.66%). Notably, the abundance of *Actinobacteriota* (14.91%) was significantly higher in the HLP group. In the FB group, the abundance of *Firmicutes* (46.57%) was similar to the CON group, while *Desulfobacterota* (6.46%) showed a significant increase (Figure 5C). At the genus level, the five most abundant genera in the CON group were unclassified_*Muribaculaceae* (16.57%), *Ileibacterium* (12.60%), *Ligilactobacillus* (10.88%), *Bifidobacterium* (7.92%), and *Muribaculum* (7.08%). Compared with the CON group, the UA group showed a marked augmentation in *Ligilactobacillus* (35.46%), and substantial decrease in unclassified_*Muribaculaceae* (11.45%), *Bifidobacterium* (1.15%), *Muribaculum* (1.80%), and *Ileibacterium* (0.05%). Following probiotic intervention, *Ligilactobacillus* (HLP group: 31.48%; LLP group: 29.40%) decreased after probiotic intervention. Apart from that, *Bifidobacterium* (13.64%) increased significantly in the HLP group. In the FB group, the abundance of unclassified_*Muribaculaceae* (18.30%) and *Ligilactobacillus* (8.96%) was similar to the CON group, while *Ileibacterium* (0.44%) and *Bifidobacterium* (0.40%) were extremely low (Figure 5D). Linear discriminant analysis (LDA) and cladogram graph revealed a significant increase in the relative abundance of *Ligilactobacillus* in UA group. High-dose *L. paracasei* N1115 significantly increased the abundance of *Bifidobacterium, Lacticaseibacillus*, and *Allobaculum* and other genera (Figures 5E, F). Ultimately, heat map was generated to showcase the composition of gut microbiota species (Figure 5G).

### 3.7 The impact of *L. paracasei* N1115 on the renal metabolism

**Figure 6:**
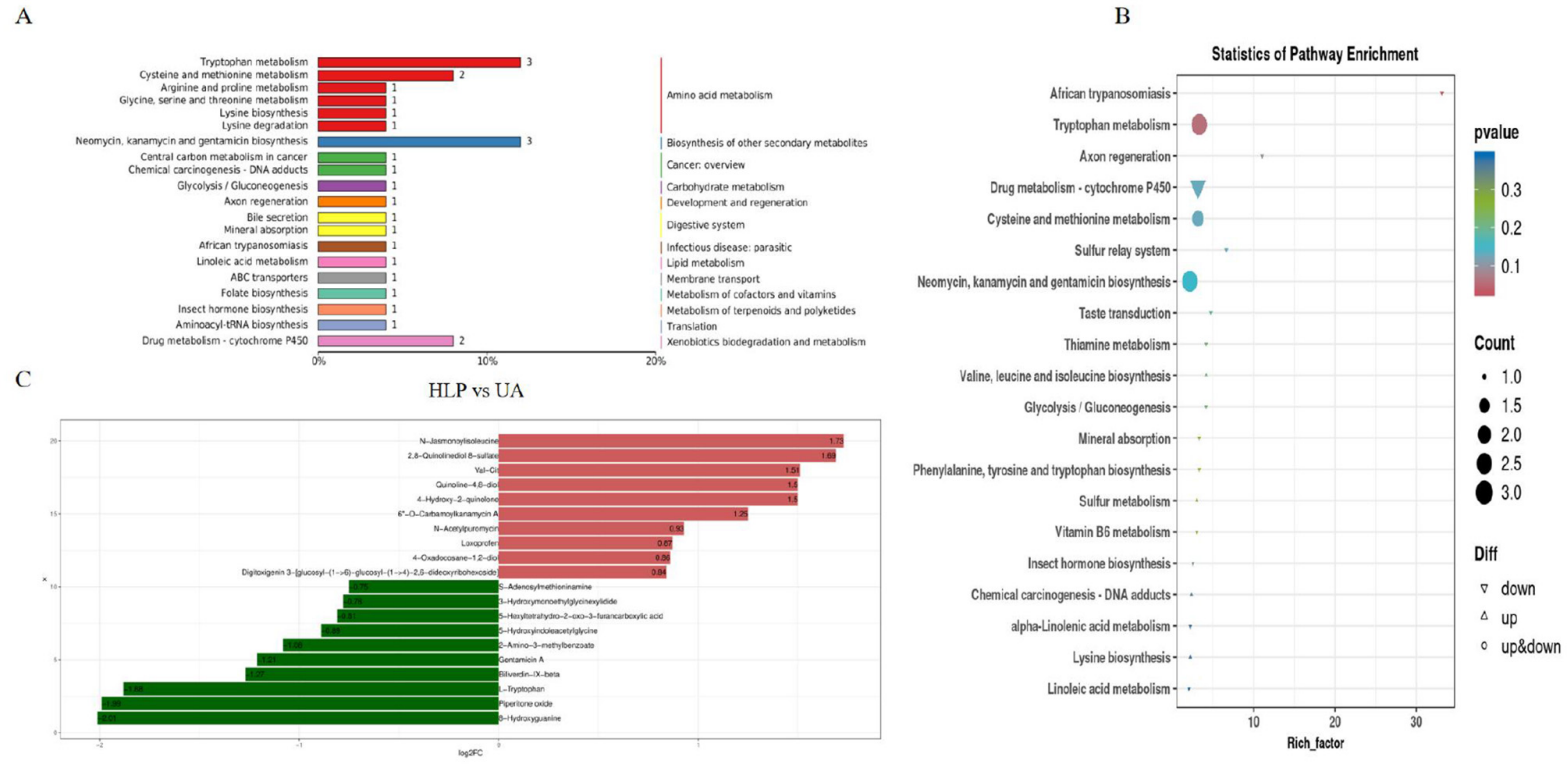
Effects of *L. paracasei* N1115 on the renal metabolism in HUA mice. **(A)** Pathway classification map of differential metabolites. **(B)** KEGG enrichment map of differential metabolites. **(C)** Fold change bar chart.

In order to explore the effect of *L. paracasei* N1115 on renal metabolism, an untargeted metabolomics analysis was conducted on the kidney samples. Differential metabolite analysis of UA group and HLP group was performed, and the pathway enrichment analysis identified significant enrichment in the tryptophan metabolism, Cysteine and methionine metabolism as well as the neomycin, kanamycin, and gentamicin biosynthesis between the UA and HLP groups (Figures 6A, B). Then the fold change bar chart reveals that the content of L-Tryptophan, Piperitone oxide, Gentamicin A (the presence of Gentamicin A may due to misannotation or contamination during LC-MS untargeted profiling as normally it is not an endogenous metabolite) in HLP group significantly elevated compared with UA group (Figure 6C). L-Tryptophan is an essential amino acid that plays a vital role in numerous physiological processes (24). Moreover, previous studies have demonstrated that tryptophan metabolism is closely associated with HUA (16).

### 3.8 *L. paracasei* N1115 modulated the generation of SCFAs in gut

Ultimately, gas chromatography was used to analyze the impact of *L. paracasei* N1115 on SCFAs in mouse gut. Owing to misoperation, only four data were obtained in the CON group. To unify the sample number, we randomly selected four out of six samples from other groups for analysis. As illustrated in Figures 2K–P, the SCFAs levels in the UA group were markedly reduced, particularly acetic acid, propionic acid, and isobutyric acid, which decreased by 58.14%, 47.13%, and 25.95%, respectively (*p* < 0.05). *L. paracasei* N1115 significantly improved SCFA levels in the intestine; however, both high-dose and low-dose treatments still exhibited a considerably low levels in SCFAs than that of the CON group, particularly in acetic and propionic acid (*p* < 0.05). Notably, compared with the UA group, butyric and isobutyric acid levels were significantly elevated (*p* < 0.05), with butyric acid levels surpassing those found in healthy control mice (*p* > 0.05). Butyrate is known to play an active role in UA metabolism (13). Furthermore, gut microbiota analysis revealed a substantial increase in the abundance of *Bifidobacterium* following probiotic intervention, which is typically positively correlated with butyrate production (25). These findings reveal that *L. paracasei* N1115 may promote UA excretion by enhancing the abundance of butyrate-producing bacteria.

## 4 Discussion

The primary objective of this study was to evaluate the UA-lowering potential of *L. paracasei* N1115. After confirming *in vitro* that its metabolites were able to inhibit XOD, animal experiments were subsequently conducted, with both high- and low-dose groups established to examine whether dosage influenced its efficacy, as we hypothesized that a higher dose of probiotics would exert a stronger effect on HUA intervention (17). It was observed that both high- and low-dose groups significantly reduced the SUA in the mice by approximately 30%, with an effect comparable to that of febuxostat. Although both HLP and LLP groups can effectively alleviate HUA in mice, no significant differences were observed between these groups in terms of regulating SUA, BUN, Cr, and most inflammatory factors. We propose two possible explanations for this phenomenon. First, the intervention period may have been sufficiently long to allow the low-dose group to regulate UA metabolism. Second, the dosage administered to the low-dose group might have already been relatively high. Normally, HUA leads to obesity (26). However, in this study, HUA resulted in weight loss in mice. This may be due to HUA damaging the intestinal barrier, which reduced energy absorption (27). Following probiotic intervention, the low body weight of HUA-affected mice increased considerably in both HLP and LLP groups. Furthermore, HUA-induced tissue damage in the liver, kidneys, and duodenum was effectively mitigated by *L. paracasei* N1115. BUN and Cr are widely considered indicators of renal function, and their reduction reflects the protective effect of *L. paracasei* N1115 on kidney function (22).

Moreover, HUA is frequently accompanied by inflammatory responses. Studies have shown that UA crystals can bind to Toll-like receptors (TLR), which are essential membrane-bound receptors in innate immunity, thereby inducing inflammation (28). Specifically, TLR-2, TLR-4, and myeloid differentiation primary response protein 88 (MyD88) are crucial for the inflammatory response of macrophages to UA crystals. These crystals directly interact with these receptors, initiating signal transduction pathways that ultimately activate the nuclear factor kappa B (NF-κB) pathway, inducing the production of various pro-inflammatory factors such as IL-1β (29). Notably, after treatment with *L. paracasei* N1115, these pro-inflammatory factors tended to return to normal levels, and the expression of the anti-inflammatory factor IL-10 is elevated compared with the UA group, effectively alleviating inflammation, consistent with previous reports on other *L. paracasei* (30, 31). It is believed that this attenuation of inflammation is closely associated with the increased butyrate content in the gut. Butyrate can act as a ligand for peroxisome proliferator-activated receptor gamma (PPARγ), thereby suppressing NF-κB activity and simultaneously upregulating anti-inflammatory cytokines such as IL-10, ultimately alleviating inflammatory responses (32). However, whether a “*L. paracasei* N1115-butyrate-inflammation axis” exists remains to be confirmed in future studies.

Additionaly, *L. paracasei* N1115 can modulate HUA by effecting UA metabolism. UA metabolism mainly contains two main parts: its production and its excretion. Both processes must be harmonized to ensure the balance of UA metabolism. In humans, UA is the final product of purine metabolism, catalyzed by different enzymes at various stages (19). 5′-NT, ADA, and XOD are key catalysts participating in UA production. 5′-NT has low substrate specificity and can catalyze the hydrolysis of hypoxanthine nucleotide and adenylate to generate corresponding nucleosides (23). ADA participates in the deamination of adenosine to form hypoxanthine nucleoside (33, 34). After the conversion of hypoxanthine nucleoside to hypoxanthine, the product is oxidized to xanthine under the participation of XOD, and then further oxidized to UA (35). Among these enzymes, XOD has emerged as a drug target for the treatment of HUA (36). *In vitro* experiments showcased that the metabolic products of *L. paracasei* N1115 has a strong inhibitory effect to XOD. Plus, *in vivo* studies also demonstrated that *L. paracasei* N1115 can effectively reduce the activity of ADA and XOD in mice as well as the content of 5′-NT, in a manner similar to febuxostat. These findings suggest that *L. paracasei* N1115 may inhibit the activity or content of enzymes related to UA production through its metabolic products. The extent of the decrease in enzyme activity is comparable to previous reports on other *L. paracasei* (31, 37). However, the present study only demonstrated the inhibitory effects of these metabolites through *in vitro* and *in vivo* characterization. The specific metabolites responsible for inhibiting UA-producing enzymes have yet to be identified and need to be confirmed in future studies.

UA excretion also plays an equally important role in UA metabolism. Renal excretion accounts for approximately two-thirds of UA elimination, mediated by multiple several transport proteins including URAT1, GLUT9, OAT1, OAT3, and NPT1. URAT1 acts as a urate reabsorption transporter, reclaiming about 90% of filtered urate back into the bloodstream following glomerular filtration. GLUT9 facilitates the trans-epithelial urate reabsorption by mediating basolateral efflux of UA into systemic circulation, thus consistently exerting inhibitory effects on urate excretion. OAT1 and OAT3, located on the basolateral membrane of the proximal tubule, function as urate/dicarboxylate exchangers responsible for UA excretion. NPT1, which exhibits a weak to moderate correlation with altered SUA levels, facilitates both the absorption and efflux of UA. It participates in sodium-phosphate cotransport (29, 38). Notably, studies have shown that *L. paracasei* N1115 effectively downregulates URAT1 and GLUT9 expression while upregulating OAT3 levels. Overall, the regulatory capacity of the HLP group was stronger than that of the LLP group on those transporters. However, this probiotic strain did not affect the expression of OAT1 or NPT1. These findings suggest that *L. paracasei* N1115 may modulate the expression of URAT1, GLUT9, and OAT3 through a specific signaling pathway, ultimately enhancing renal urate excretion and reducing systemic reabsorption. This collective effect likely contributes to the reduction of serum UA levels in mice models (Figure 4). Existing animal experimental evidence has indicated that *L. paracasei* can lower UA levels in HUA mice by modulating urate transporters. For example, *L. paracasei* MJM60396 has been reported to upregulate OAT1 and OAT3, while downregulating URAT1 and GLUT9 (11). Additionally, intervention with *L. paracasei* LT12 significantly downregulated URAT1 and GLUT9, while upregulating OAT1 and ABCG2 (37). These findings are broadly consistent with the results obtained in our study. However, reports explaining the specific molecular mechanisms remain limited. In study evaluating the UA-lowering potential of *L. paracasei* LT12, an increase in butyrate levels was also observed (37). Previous studies have also found that supplementation with butyrate alone can modulate certain transporters, such as GLUT9 and ABCG2 (39). Future studies could incorporate metabolomics of *L. paracasei* to further explore the specific molecular mechanisms underlying its regulation of transporters.

Although no particularly significant differences were observed between the high- and low-dose groups in most parameters, we found that they differed in shaping the intestinal microbiota. The gut microbiota exhibits a complex interaction UA metabolism. On one hand, UA functions as both an antioxidant and immunomodulatory agent, significantly influencing the composition of the gut microbiota (40, 41). Conversely, the gastrointestinal tract plays a crucial role in UA excretion, with the gut microbial ecosystem actively contributing to this metabolic process (42). UA is transported into the intestinal lumen via specific transporter proteins, where it undergoes either direct excretion or microbial-mediated decomposition (43–45). This study demonstrated that excessive UA accumulation disrupted gut microbiota structure. The *L. paracasei* N1115 increased the microbiota richness of HUA mice. Unexpectedly, differential effects were observed between high-dose and low-dose *L. paracasei* N1115 on microbial community abundance. High-dose administration resulted in a decreased species richness compared with low-dose treatment (Figure 5A). This may be attributed to high-dose probiotics competing with native gut microorganisms for limited resources, such as nutrients and space, thereby inhibiting the growth and reproduction of some native species and reducing overall microbial richness. However, the potential side effects of this phenomenon remain undetermined. Overall, after *L. paracasei* N1115 intervention, the abundance of *Bifidobacterium* increased significantly, and *Ligilactobacillus* levels were also higher compared with those in healthy control mice. Previous literature has also reported that certain strains of *L. paracasei*, such as *L. paracasei* JY062 and *L. paracasei* L9, can increase the relative abundance of *Bifidobacterium* (46, 47). *Lactobacillus* and *Bifidobacterium* have a cross-feeding relationship in the gut. *Bifidobacterium* uses *Lactobacillus*-produced lactic acid and other metabolites as growth substrates (48). Previous studies have indicated that *Bifidobacterium* is negatively correlated with SUA (49). Additionally, some *Ligilactobacillus* can directly degrade purine compounds, thereby reducing UA production (50). Apart from that, *Ligilactobacillus* generates lactic acid, which helps maintain the acidic environment of the gut to inhibit the growth of harmful bacteria. Plus, it plays a role in enhancing the immune system and improving gut barrier integrity as well (51, 52). Notably, after high-dose intervention, the abundance of *Allobaculum* also increased. Although the research on *Allobaculum* is less extensive than that on other probiotics, studies have shown that it can help maintain gut health (53).

Following *L. paracasei* N1115 intervention, not only is microbial diversity improved, but the production of specific SCFAs, such as butyric acid, is also enhanced (Figures 2M, N). SCFAs are believed to play a key role in UA metabolism and serve as crucial factors of the kidney-gut axis. For instance, acetate can lower the intestinal pH, inhibit the growth of harmful bacteria, and strengthen the intestinal barrier. Once absorbed in the small intestine, acetate circulates throughout the body and is able to inhibit XOD activity in the kidneys and bloodstream (14). As mentioned before, butyrate, another SCFAs, regulates the expression of the transporter protein ABCG2 and GLUT9, which are widely distributed in both the gut and the kidneys (13, 39). Consequently, the gut microbiota can influence renal UA metabolism through the butyrate pathway. Additionally, butyrate exhibits anti-inflammatory and immunoregulatory properties by inhibiting the activation of the NF-κB pathway, thus mitigating systemic inflammation—an effect particularly important for maintaining UA homeostasis and the health of kidney-gut axis (54, 55). Numerous studies have reported that *L. paracasei* can increase intestinal butyrate levels (56). However, it is noteworthy that *L. paracasei* itself is unable to produce butyrate, indicating that the source of butyrate warrants further attention. In the present study, we observed that *L. paracasei* N1115 administration, particularly in the high-dose group, markedly increased the abundance of *Bifidobacterium*. This may be attributed to a cross-feeding relationship between *Bifidobacterium* and *L. paracasei*, as noted (48). Previous studies have demonstrated a positive correlation between the abundance of *Bifidobacterium* and intestinal butyrate levels (57). The metabolites of *Bifidobacterium* can serve as prebiotics for butyrate-producing bacteria, such as *Anaerostipes caccae* and *Roseburia intestinalis*, thereby enhancing intestinal butyrate concentrations (25). Therefore, our findings suggest that *L. paracasei* N1115 may alleviate HUA by increasing butyrate levels through a cross-feeding relationship with *Bifidobacterium*, which warrants further validation in future studies.

In addition to the SCFAs regulation, this study demonstrated that *L. paracasei* N1115 also exerts a pronounced impact on renal metabolic pathways, especially that of tryptophan metabolism (Figures 6A, B). Even if the present experiments could not fully substantiate underlying mechanisms of this regulation, the hypothesis based on the studies and existing papers is as followed: *Bifidobacterium* can enhance the production of tryptophan metabolites, especially indole-3-lactic acid (ILA), an intermediate in IPA production (58, 59). As a consequence, *Bifidobacterium* is strongly associated with the elevation of IPA level (60, 61). IPA can activate the aryl hydrocarbon receptor (AhR), thereby suppressing inflammation associated with NLRP3/GSDMD pyroptosis in the colon. Furthermore, IPA can upregulate ABCG2 expression, facilitating UA excretion (16, 62). These suggest that *L. paracasei* N1115 may indirectly modulate UA metabolism via influencing *Bifidobacterium*, leading to enhanced expression of transporter proteins such as ABCG2 and regulation of other related metabolism pathways, such as tryptophan metabolism. Therefore, further experimental studies will be arranged to explore this hypothesis.

## 5 Conclusion

The experimental results demonstrate that *L. paracasei* N1115 can alleviate HUA in mice. Firstly, *in vitro* experiments showed that the metabolic products of *L. paracasei* N1115 has a strong inhibitory effect to XOD. *In vivo* experiments, *L. paracasei* N1115 intervention reduced SUA, protected kidney function, mitigated tissue damages and inflammation induced by HUA. Additionally, it effected UA production by modulating enzymes related to UA synthesis, including 5′-NT, ADA, and XOD. *L. paracasei* N1115 also promoted UA excretion by reducing the urate reabsorption proteins GLUT9 and URAT1 and enhancing the UA excretion protein OAT3. Futhermore, *L. paracasei* N1115 reshaped the gut microbiota and significantly increased the abundance of *Bifidobacterium*, while modulating renal metabolism elevating butyric acid levels in gut. These findings suggest that *L. paracasei* N1115 may improve the urate metabolism via enhancing butyric acid levels through cross-feeding relationship with *Bifidobacterium*. Although further experiments are required to substantiate underlying mechanisms, this study provides a basis for anti-HUA functional foods development.

## Data Availability

The datasets presented in this study have been deposited to the NCBI (https://www.ncbi.nlm.nih.gov) with the accession number PRJNA1329173. Further inquiries can be directed to the corresponding authors.
